# Recent Trends in Structures and Interfaces of MEMS Transducers for Audio Applications: A Review

**DOI:** 10.3390/mi14040847

**Published:** 2023-04-14

**Authors:** Alessandro Gemelli, Marco Tambussi, Samuele Fusetto, Antonio Aprile, Elisabetta Moisello, Edoardo Bonizzoni, Piero Malcovati

**Affiliations:** Department of Electrical, Computer and Biomedical Engineering, University of Pavia, 27100 Pavia, Italy; alessandro.gemelli02@universitadipavia.it (A.G.); marco.tambussi01@universitadipavia.it (M.T.); samuele.fusetto01@universitadipavia.it (S.F.); antonio.aprile01@universitadipavia.it (A.A.); piero.malcovati@unipv.it (P.M.)

**Keywords:** MEMS, microphones, speakers, structures, electromagnetic, electrostatic, piezoelectric, ICs, interfaces, trends

## Abstract

In recent years, Micro-Electro-Mechanical Systems (MEMS) technology has had an impressive impact in the field of acoustic transducers, allowing the development of smart, low-cost, and compact audio systems that are employed in a wide variety of highly topical applications (consumer devices, medical equipment, automotive systems, and many more). This review, besides analyzing the main integrated sound transduction principles typically exploited, surveys the current State-of-the-Art scenario, presenting the recent performance advances and trends of MEMS microphones and speakers. In addition, the interface Integrated Circuits (ICs) needed to properly read the sensed signals or, on the other hand, to drive the actuation structures are addressed with the aim of offering a complete overview of the currently adopted solutions.

## 1. Introduction

Integrated audio systems are a hot topic within the current technology market; precise sound sensing, processing, and generation are required for several cutting-edge applications that are progressively becoming part of our lives. In this framework, MEMS devices are widely employed as transducers thanks to their compatibility with standard CMOS (Complementary Metal Oxide Semiconductor) processes (typically adopted for the signal processing chain), low cost, and compactness; accordingly, the growth of the MEMS manufacturing technology has given rise to significant advances regarding microphones and speakers, the focus of this review.

MEMS microphones are widely employed in mobile phones [1,2,3,4,5,6,7,8,9,10,11] and wearable devices [1,2,5,6,12,13] to capture high-quality audio for calls and recordings, whereas in the automotive field [10,14,15,16], they are used for hands-free calling, voice control, or even pedestrian detection [17]. They are also exploited for medical applications [13,14,18] such as smart stethoscopes [19,20], blood pressure monitoring, or to detect abnormal heartbeats [21]; in addition, these devices are a full-fledged part of the Internet of Things (IoT) world [2,5,18,22]. Currently, one of the applications of major interest in the consumer market is Voice Activity Detection (VAD), which exploits voice as a vector for human/machine interface; indeed, MEMS microphones are employed within smart voice assistants [7,10,23,24] such as Amazon Alexa and Google Home that operate according to the user’s voice commands, are embedded in the remote controls of smart TVs, and could even be installed in the rooms of smart homes [5]. In addition, they are used in the Augmented Reality (AR) and Virtual Reality (VR) fields [25,26,27] to enable communication with state-of-the-art Head Mounted Displays (HMD), while in the True Wireless Stereo (TWS) framework, they are exploited to achieve a crystal-clear voice pick-up through beamforming and noise cancellation techniques. Lastly, they are increasingly being used for cochlear implants [28,29,30] and hearing aids [1,4,8,10,16,24,31,32,33].

On the other hand, MEMS speakers are running through a time of remarkable growth. Their development is mainly driven by the need for thinner and thinner devices embedding sound actuators with reduced volume occupation to achieve improved cost-effectiveness. Moreover, considering that this kind of device is widely employed for a series of applications that are battery operated (smartphones [34,35,36,37,38,39,40,41,42], smartwatches [37,43,44], IoT [35,36,43,45], etc.), another significant trend concerns their energy efficiency; indeed, their development is oriented to maximize the output acoustic power for a given electrical power consumption. MEMS speakers are extensively adopted in hearing aids to provide sound amplification for people with hearing loss [34,35,36,37,38,40,41,46,47,48,49] or even as part of a fully implantable cochlea [50]. In headphones [34,36,38,43,46,51] and earbuds [35,36,39,40,44,52], they are employed to provide high-fidelity and low distortion audio playback, whereas in the medical field, they are used for health warning applications [44]. Moreover, MEMS audio actuators could be employed in acoustic fluidics applications in order to enable matter manipulation in the miniaturization domain, e.g., in lab-on-chip solutions, nanoparticle patterning, or single cell studies [53,54].

**Figure 1:**
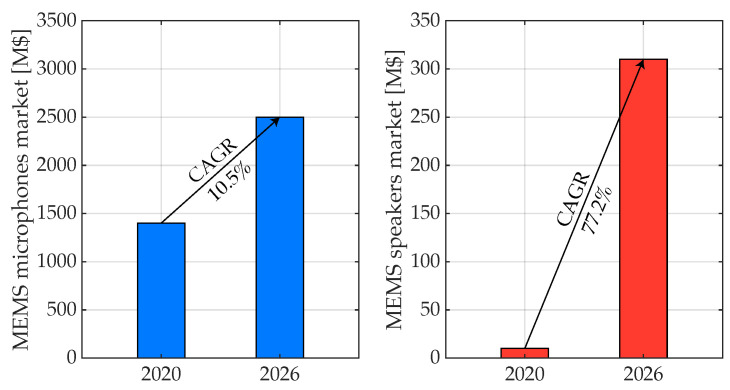
MEMS microphones and speakers market forecast.

To further highlight the evolution of the MEMS transducers taken into account, it is interesting to consider the market forecast reported in Figure 1.

According to [55], in the next years, the market trend for MEMS microphones (left) is expected to be significantly rising, while the MEMS speakers one (right), which currently exhibits 10× lower revenues, is expected to be even booming; considering the 2020 to 2026 time interval, the Compound Annual Grow Rate (CAGR) of the first amounts to 10.5%, while the second one is as high as 77.2%. This further confirms the relevance of the proposed review, which is organized as follows. Section 2 is entirely devoted to an extensive analysis of the various transduction principles adopted so far with special attention to the structure of these micromachined devices, while Section 3 presents a detailed state-of-the-art investigation that shows the evolution of both MEMS microphones and speakers across the last 15 years. Moreover, Section 4 is focused on the interface circuits typically employed as readout for the sensed signal and, on the other hand, to drive the actuation devices; Section 5 concludes the review by recalling its focal points and providing a global overview of the derived trends.

## 2. Transduction Principles

MEMS speakers and microphones may rely on different transduction principles in order to implement actuation and sensing: the exploited mechanisms are electromagnetic (EM), electrostatic, piezoelectric, piezoresistive, optical, spintronic, and thermoacoustic. The electromagnetic, electrostatic, and piezoelectric/piezoresistive principles are employed both for actuation and sensing; optical and spintronic methods are adopted only for microphones, while thermoacoustic transduction is used only for speakers.

Electromagnetic transducers (also known as electrodynamic), consisting of a magnet, a coil, a diaphragm, and a structure to provide support and enclosure, rely on the Lorentz force for their working principle. In microphones, the coil, attached to the diaphragm, which vibrates according to the acoustic input, moves through the fixed magnetic field determined by the magnet, thus producing an alternate current as output. In speakers, the permanent magnet moves together with the diaphragm, to which it is attached, while the coil is fixed; when current flows through the coil, the generated force actuates the diaphragm, determining its movement and therefore sound emission.

Electromagnetic microphones feature low noise, low-impedance and do not require pre-amplifiers; however, they suffer from low sensitivity due to the slow vibration velocity caused by the heaviness of the diaphragm and coil [33].

**Figure 2:**
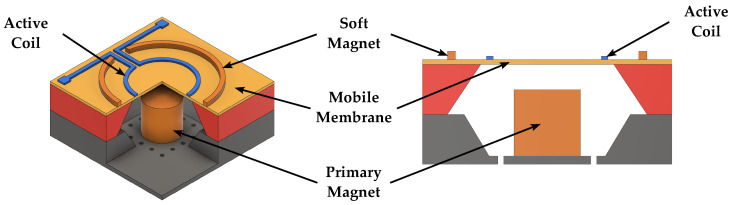
Schematic 3D view and cross section of an electromagnetic speaker employing the structure proposed in [41].

**Figure 3:**
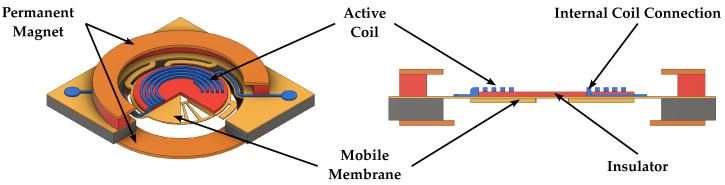
Schematic 3D view and cross section of an electromagnetic speaker employing the structure proposed in [42].

Electromagnetic speakers, used in most consumer electronics applications, can provide optimal acoustic performance, especially in terms of linearity and high-fidelity sound reproduction, while employing low driving voltages, thanks to their larger driving force and subsequent diaphragm displacement. However, their fabrication and packaging are quite expensive as the assembly of the magnet is generally required, thus adding additional challenges to the MEMS process [41,42,48,49,56]. Schematic 3D representations and cross sections of electromagnetic MEMS speakers are reported in Figure 2 and Figure 3.

Electrostatic transducers are based on a flexible diaphragm as a movable electrode and a rigid backplate with acoustic vent holes as a fixed electrode; the two conductive plates acting as electrodes are placed in a parallel geometric arrangement, separated by an air gap [57]. Alternatively, the diaphragm can be rigid and supported by springs, which, by bending, enable the device’s movable electrode plate to achieve piston-like motion [7,42].

**Figure 4:**
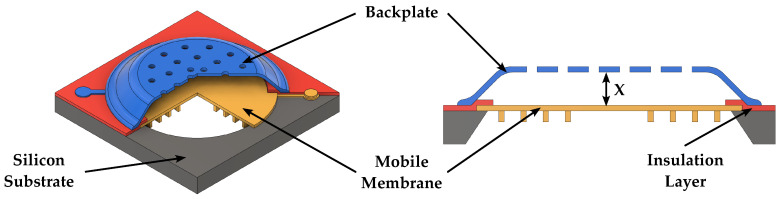
Schematic 3D view and cross section of a capacitive microphone.

In a microphone, the diaphragm implementing the movable plate deflects according to the acoustic pressure, thus varying its distance from the fixed plate and, therefore, the capacitance value implemented by the parallel plates, which need to be biased at a fixed voltage. The bias voltage between the plates can be provided by a voltage source or by means of an electret material [56]. Electrostatic microphones are well compatible with batch fabrication MEMS processes, thus allowing low-cost production; however, they require high bias voltages. The 3D sketch and the cross section of a capacitive microphone are illustrated in Figure 4.

In electrostatic speakers, the diaphragm movement, and therefore the sound output, is determined by the force generated by the electrostatic field between the plates under AC voltage driving [47]. Although they feature high miniaturization and cost-effective fabrication, electrostatic speakers suffer from non-linearity issues, are limited by the pull-in effect, and require high driving voltages [39,58,59].

**Figure 5:**
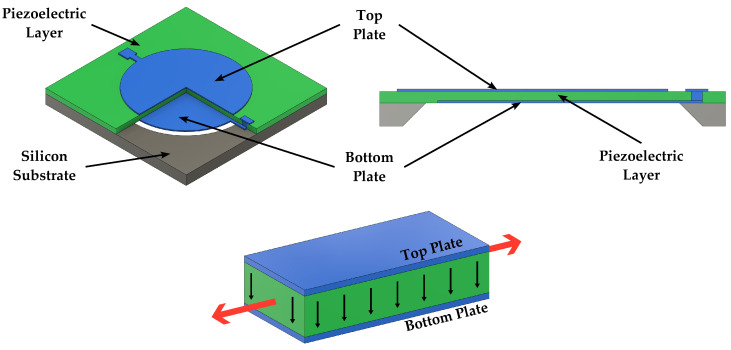
Schematic 3D view and cross section of a piezoelectric speaker exploiting the $d_{31}$ mode.

**Figure 6:**
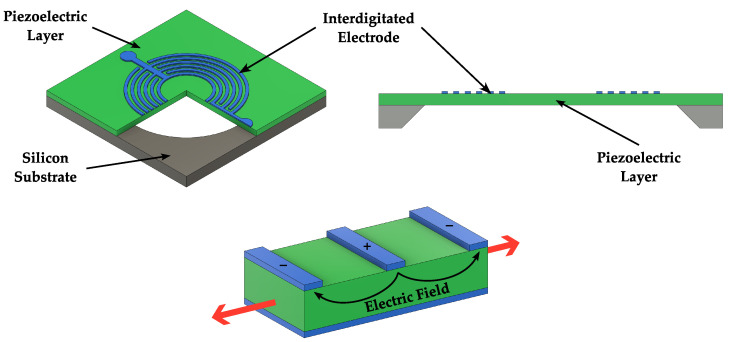
Schematic 3D view and cross section of a piezoelectric speaker exploiting the $d_{33}$ mode.

Piezoelectric transducers feature, in addition to at least a pair of electrodes, a single flexible plate without the need for a fixed backplate. They rely on the piezoelectricity mechanism to convert mechanical vibrations, and hence sound, into electrical signals and vice versa [10,60]. Energy conversion occurs according to two transducing modes depending on the electrodes arrangement: $d_{31}$ mode or $d_{33}$ mode, where the pedices indicate the direction of the polarization and the strain. As illustrated in Figure 5, the $d_{31}$ mode typically implies the use of top and bottom electrodes, while the $d_{33}$ mode, as shown in Figure 6, employs interdigitated electrode structures [13,61]. As the transducer performance is dependent on the electrodes spacing, the $d_{33}$ mode allows more design freedom than the $d_{31}$ mode as the distance between the electrodes is no longer directly dependent on the piezoelectric material film thickness [13]. Silicon does not feature piezoelectric properties; hence, it must be integrated with appropriate piezoelectric materials (e.g., PZT, AlN, and ZnO) in order to achieve the desired electromechanical transduction. This may increase the complexity of the MEMS fabrication process, ultimately limiting the achievable devices’ performance [14,40]. Nevertheless, since process technologies and material properties for piezoelectric thin films have been continuously improving in recent years, this could lead to significant performance enhancements [40]. Concerning the process complexity, however, as only one membrane with no backplate is required, manufacturing and costs are in a way simplified [10]. Since piezoelectric sensing is passive, microphones do not require any voltage biasing, thus enabling very low power consumption [18]. Furthermore, due to the absence of an air gap, piezoelectric microphones are relatively robust against dust and particles and their detrimental effect on sensitivity and Signal-to-Noise Ratio (SNR) [10].

**Figure 7:**
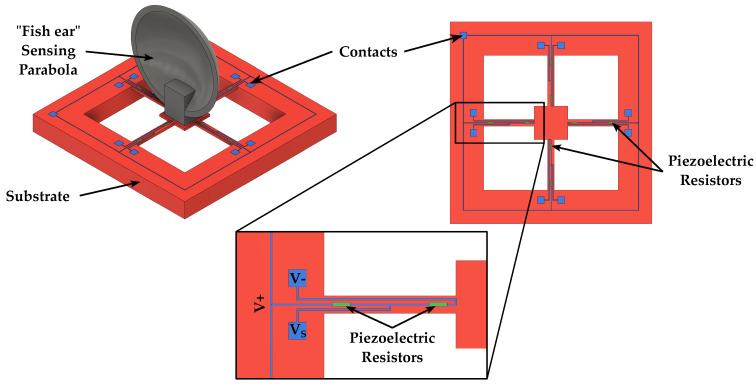
Schematic 3D view and top view of a piezoresistive microphone based on the “fish ear” structure proposed in [20].

A variation of the piezoelectric microphone is the piezoresistive microphone, which features four resistors connected in a Wheatstone bridge on top of the flexible membrane. When the diaphragm deflects in response to the pressure induced by sound waves, the stress-dependent values of the resistors change accordingly, and the Wheatstone bridge produces an output voltage based on the difference between the values of these resistors. A 3D schematic view of a piezoresistive microphone, featuring in addition the “fish ear” structure proposed in [20], is illustrated in Figure 7. With respect to typical piezoelectric microphones, the piezoresistive microphone features lower dynamic range and sensitivity [56].

Piezoelectric speakers offer advantages in terms of low driving currents and voltages, resulting in low power consumption, low cost, and very small size [39,40,59,62]. However, due to residual stress and charge leakage inside the material, piezoelectric transducers, suffer from poor performance at low frequencies [14,63].

**Figure 8:**
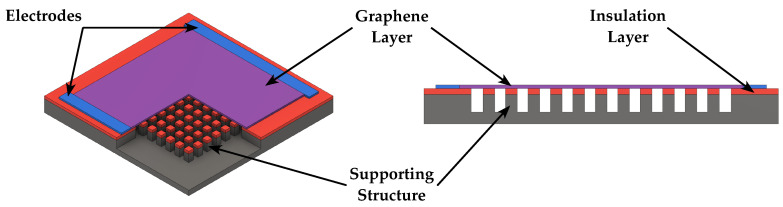
Schematic 3D view and cross section of a thermoacoustic speaker.

Speakers feature an additional transduction mechanism based on the thermoacoustic effect, which transforms the Joule heat of conductors into sound. While previously discussed actuation principles relied on the mechanical vibration of the diaphragm to produce pressure waves, in this case sound is produced by the periodic contraction and expansion of the medium (typically air) around the diaphragm, determined by the thermal energy exchange between the diaphragm and the surrounding medium when the diaphragm is heated by applying an AC current [52,64,65]. Thermoacoustic speakers, typically made of carbon nanotubes or graphene films, feature a simple and light weight structure, resulting in easy fabrication. Current thermoacoustic speakers, however, require a size in the few centimeters range and high power consumption, going from 100 mW to a few watts, in order to produce a sufficient sound pressure. Nevertheless, as graphene and carbon nanotubes can be transparent (when their size is in the few nm range) and fabricated into any shape and size due to their stretchable nature, either on an insulating surface or freestanding, they show great potential for developing thermoacoustic MEMS speakers [52]. A 3D schematic representation of a thermoacoustic speaker is illustrated in Figure 8.

MEMS microphones may rely on two additional sensing principles: spintronic and optical mechanisms. The spintronic microphone aims at solving the low sensitivity problem found in piezoresistive microphones by substituting the resistors on the acoustic diaphragm with spin strain gauge sensors, thus implementing a magneto-resistance transduction mechanism [66,67,68,69]. Optical or fiber-optic microphones, instead, detect deflections induced by sound in the diaphragm thanks to light intensity modulation: a light source, usually a laser diode, is used to illuminate the reflective diaphragm, while an optical sensor, typically a photodiode array, is employed for detecting the light’s wavelength and intensity; hence, when the membrane vibrates according to the sound waves, the difference between the original light source and the reflected one is recorded and converted into an electrical signal. Optical microphones are not susceptible to electronic noise, thus featuring high SNR, and are robust against electromagnetic interference. They are, however, very expensive due to the complexity of the detection system as well as significantly power hungry; for these reasons, they are more tailored for high-end applications where power consumption and cost are not a concern [10,56].

Although thermoacoustic actuation, spintronics, and optical sensing feature interesting properties and look promising for future developments in MEMS audio transducers, they are not yet mature and are still in the initial phases of research. For this reason, only electromagnetic, electrostatic and piezoelectric/piezoresistive devices are considered in this review when analyzing the State-of-the-Art in Section 3.

## 3. State-of-the-Art

Taking the previously introduced transduction types into account, it is interesting to compare the large amount of published works over the past 15 years in order to extract the research trends and derive future perspectives regarding MEMS audio devices. This section presents a detailed state-of-the-art analysis for both microphones and speakers, highlighting their features according to the employed materials and operation principles.

### 3.1. MEMS Microphones State-of-the-Art

Although MEMS microphones can be implemented by exploiting the electromagnetic sensing principle [33], the added complication to the fabrication process given by the need to integrate magnetic materials has led researchers to focus almost exclusively on MEMS electrostatic (capacitive) or piezoelectric sensing solutions.

**Figure 9:**
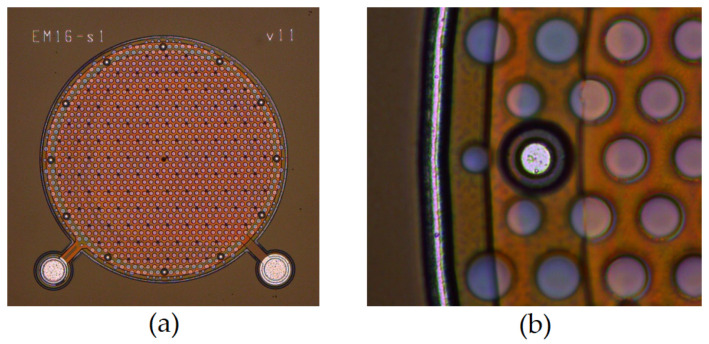
Microscope image of (**a**) the fabricated capacitive MEMS microphone in [11], with (**b**) an enlarged view of the dual-anchor structure.

Capacitive devices, in particular, were the first MEMS microphones to have been investigated, and now represent the majority of commercial MEMS microphone solutions. Indeed, they require simple fabrication processes compatible with standard CMOS technology, which enable large-volume and low-cost production. A single-crystalline silicon-based process, requiring only two photolithography steps and two wet-etching steps, was employed in [70] to monolithically fabricate the complete microphone. A dual-anchored MEMS microphone, which does not require any additional processing or mask, was proposed in [11]; the capacitive device is reported in Figure 9. Two polysilicon-layer micromachining processes, providing excellent temperature stability and compatibility with solder reflow, were also employed [4]. In order to further simplify the fabrication process, the KOH (potassium hydroxide) etching steps needed to realize the back chamber and perforated backplate were avoided in [32,71] by creating the holes for reducing acoustical damping in the microphones directly on the diaphragm. In order to reduce the diaphragm deformation by residual stress and increase the sensitivity and the SNR in capacitive microphones, a piston-like motion of the two parallel plates can be achieved by employing a rigid diaphragm supported by springs [4,7]. Moreover, an SNR and sensitivity improvement can be achieved by increasing the effective area of the diaphragm by employing peripheral and central protrusions on the backplate [16].

Graphene-based membranes have also been investigated in recent years, as graphene features a low mass density, which is an advantage when creating suspended structures, as well as the ability to form a one atom thick film, which would result in a larger membrane’s mechanical response to sound pressure [72].

In order to facilitate the fabrication of MEMS capacitive microphones, solutions without the need for a backplate were proposed by fixing the reference sensing electrodes to the substrate [6] and by using planar interdigitated electrodes that act as vertical comb sensing elements [57]. As the presence of the backplate is also a source of noise due to its acoustical resistance, removing it would also improve the SNR. An alternative approach for solving the noise issue determined by the backplate is isolating it from the membrane and performing the transduction in vacuum instead of removing it; the separation is made possible by a mechanical hinge, which is able to transmit a mechanical motion between two atmospheres [73,74].

Typical capacitive microphones suffer from decreased performance at low frequencies, due to the reduction in air gap capacitance that results from the microphone’s miniaturization. In order to overcome this limitation, the Electret Gate of Field-Effect Transistor (ElGoFET) microphone was introduced [22,75]. The ElGoFET device combines a field-effect transistor (FET), embedded in the diaphragm, and an electret; a displacement of the diaphragm due to the acoustic pressure leads to a change in the separation distance between the FET and the electret, which results in a change in the electric field across the air gap and, therefore, in a change in the FET drain-source current. As the sensitivity in ElGoFET transduction is dependent on the ratio of capacitive components in the transduction structure, high sensitivity can be achieved also at low frequency, even with a smaller air gap capacitance due to the miniaturization of the microphone [22,75].

Other than the poor performance at low frequencies, another issue in MEMS capacitive microphones is the pull-in effect, which causes the diaphragm to collapse on the fixed electrode once a certain bias level, known as the pull-in voltage, is exceeded, thus damaging the microphone. The pull-in effect limits the sensor’s performance, as bias voltages that would determine large diaphragm displacement and hence a large signal must be avoided. In order to solve this issue, planar interdigitated electrodes [57] or a levitation-based electrode configuration [76] were proposed.

Although capacitive solutions represent the majority of commercial MEMS microphones, researchers in the last years have moved their focus towards piezoelectric devices in order to overcome the need for the relatively large bias voltages required by capacitive microphones, which may limit their use in wearable and very low-power applications. Indeed, piezoelectric microphones are passive and therefore do not require biasing.

Usually employed piezoelectric materials include PZT (lead zirconate titanate) [77,78], ZnO (zinc oxide) [18,19,31] and AlN (aluminum nitride) [5,13,24,79,80,81]. PZT features high piezoelectric coefficient and thus a significant sensor output, however, it features higher noise and, as it contains lead, it is not environment-friendly: hence, other piezoelectric materials have attracted more interest in recent years. AlN, in particular, featuring a low dielectric loss tangent, appears to be a good solution for reducing noise [13] and achieving good performance.

**Figure 10:**
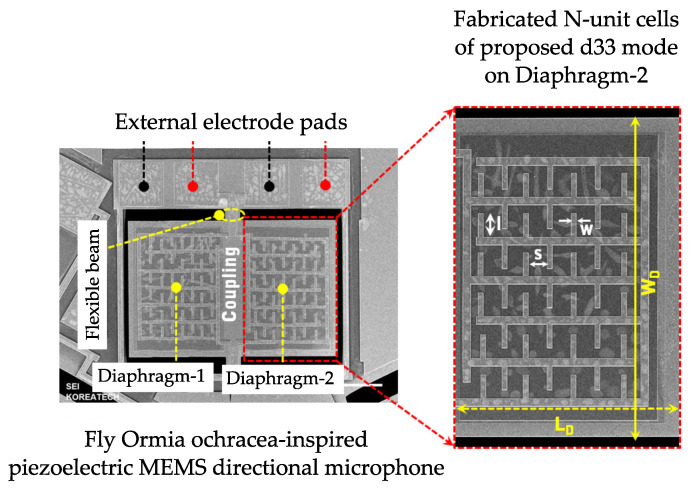
Scanning Electron Microscope (SEM) image of a fabricated biomimetic piezoelectric MEMS directional microphone [25]. $L_{D}$ (880 µm) and $W_{D}$ (1200 µm) are each individual diaphragm’s length and width, respectively. The inset shows in detail the planar interdigitated structure implemented to exploit the $d_{33}$ mode.

In order to increase the sensor performance, the employment of AlN was coupled with the use of the piezoelectric effect according to the $d_{33}$ mode, thus making the performance independent from the thickness of the piezoelectric layer and enhancing the SNR [13,79,81]. A micrograph of a device adopting this approach is shown in Figure 10.

Non-standard piezoelectric materials, such as Silicon NanoWires (SiNW), have been investigated as well; indeed SiNW feature a giant piezoresitive effect and are well suited to miniaturization [2].

Piezoelectric solutions have been widely employed for implementing microphone arrays [18,19,31]. The array consists of multiple MEMS microphones, each with a different resonant frequency: as the maximum sensitivity is obtained at resonance, by combining devices with different resonance frequencies, large sensitivity across the band of interest can be achieved; alternatively, by appropriately tailoring the resonant frequency, they can be employed as filters, such as for active noise cancellation [31].

Piezoelectricity can also be employed for energy harvesting: hence, one of the future trends in research is to use the sensing element to directly power the circuitry, as this would dramatically change the field of hearing aids, leading to a device that could be worn continuously [30].

Future trends in the MEMS microphones field include the combination of both capacitive and piezoelectric transduction mechanisms in the same device [80], the performance optimization of standard MEMS microphone designs thanks to accurate finite-element analysis [82] and the investigation of novel biomimetic structures, such as the fish ear [20], after the success obtained by directional microphones based on the hearing system of the female Ormia ochracea fly [79].

A summary of state-of-the-art MEMS microphones is reported in Table 1.

Most devices cover the so-called audio band (20 Hz–20 kHz); however, smaller bandwidths covering only the human speech spectrum (300 Hz–4 kHz) [31] or the low frequencies typical of lung wheezing or heart sounds (<1 kHz) can be encountered [18,20]. The electromagnetic microphone is significantly larger, featuring an area of 200 mm^2^ [33], while capacitive and piezoelectric device membranes can be as small as 0.071 mm^2^ [6] and 0.49 mm^2^ [31], respectively, thus providing a significant benefit in terms of miniaturization. Typical sensitivity measured at 1 kHz ranges between −45 and −35 dBV/Pa, reaching values as high as −17.2 dBV/Pa [18] or as −13.9 dBV/Pa at resonance [19]. SNR values larger than 60 dB, derived considering the sensitivity at 1 kHz, are achieved for both capacitive and piezoelectric devices, with a maximum SNR equal to 85.8 dB obtained by [18]. Overall, taking small area, good sensitivity, high SNR and large bandwidth into account, [78] appears to provide the best compromise, as it features 0.64-mm^2^ membrane size, −33.2-dBV/Pa sensitivity, 82.4-dB SNR, while covering the whole audio bandwidth.

### 3.2. MEMS Speakers State-of-the-Art

Research interest focused on MEMS speakers later on with respect to MEMS microphones; however in recent years, with the spread of IoT, wearables, and portable devices and the push towards device miniaturization, MEMS speakers have also become a hot topic. With respect to MEMS microphones, more interest has been devoted to electromagnetic-based solutions: indeed, most traditional speakers rely on electromagnetic transduction, so maintaining the same actuation principle while shrinking the device size was a natural first research step; moreover, electromagnetic speakers provide high linearity and acoustic response [34,42,48,49]. Apart from device miniaturization, the other trend in MEMS electromagnetic speakers research has been the reduction in power consumption: sub-mW performance was achieved in [34,49]. Typically, polymer diaphragms are employed in MEMS electromagnetic speakers (e.g. PDMS [48], polyimide [49]); however, alternative membrane materials have been investigated: [34] proposed a parylene/graphene/parylene composite layer membrane for bass sound and power consumption improvement, while [42] abandoned the polymer-based membrane in favor of a rigid silicon membrane suspended by highly flexible silicon springs, which allowed large out-of-plane displacement of the membrane, thus improving bass rendering and acoustic intensity over the whole bandwidth; furthermore, since silicon features low density, the mobile mass was reduced and the speaker efficiency improved as a result.

Good performance notwithstanding, MEMS electromagnetic speakers require a complex fabrication process due to the presence of the magnetic elements, which increases their cost. For this reason, alternative actuation principles have been investigated as well.

**Figure 11:**
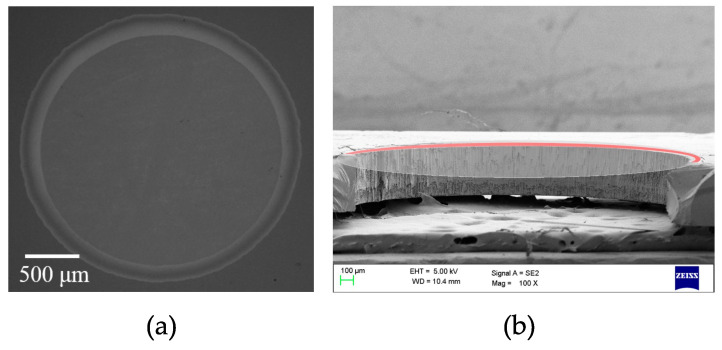
Microscope photograph of the top (**a**) and cross-section (**b**) view of a fabricated electrostatic MEMS speaker [85]. The diaphragm was removed in (**b**) for obtaining a clearer view of the structure.

Electrostatic speakers can be realized with industrial CMOS-MEMS processes with only very few additional post-process steps [47,83], obtaining frequency responses devoid of any resonance peaks [84] and achieving high linearity thanks to pre-distortion of the driving signal [58]. Figure 11 illustrates a microscope photograph of an electrostatic speaker device.

In addition, novel structures have been investigated in order to diminish damping losses and increase power efficiency: a peripheral electrode configuration was proposed in [86], and a membrane requiring no support anchors thanks to electrostatic levitation was introduced in [87].

Electrostatic devices, however, suffer from the pull-in effect, although solutions, such as an appropriate electrode configuration [86], have been proposed to increase the pull-in voltage; furthermore, they typically require rather large driving voltages [47,83,84,86]. For these reasons, the research interest has moved towards the employment of piezoelectric devices.

MEMS piezoelectric speakers feature the advantages of low driving voltages, low power consumption, and no pull-in effect. However, they inherently suffer from limited Sound Pressure Level (SPL) and low-frequency acoustic response due to their very small size. In order to increase their acoustic output, materials with a large piezoelectric coefficient like PMN-PT [61] or PZT [40,43,44,46,62,63,88,89] are employed. Ceramic PZT [39], in particular, looks promising, as its piezoelectric coefficient is even larger than that of PZT in thin film or sol-gel form. PZT, however, due to its ferroelectric properties, is non-linear [35]; moreover, its thin film deposition process is not directly compatible with standard CMOS processes, thus requiring additional process steps. For these reasons, AlN, despite featuring a lower piezoelectric coefficient, is of particular interest: its thin film deposition process is, indeed, quite mature and fully compatible with CMOS processes. Many MEMS speakers employing standard AlN were proposed [35,36,59]; moreover, scandium doping was employed as well in order to increase the piezoelectric coefficient of regular AlN [51].

In order to improve the acoustic response of MEMS speakers, not only material-wise, but also structural solutions have been investigated. A single-curve and a dual-curve spring architectures were proposed in [40] as alternatives to the traditional clamped diaphragm structure and bimorph cantilevers staking two piezoelectric layers, instead of standard unimorph cantilevers with a single piezoelectric layer, were employed in [59]. Moreover, attention was paid to the membrane sealing issue: sealed membranes [88] imply smaller membrane vibration displacement, while unsealed membranes [46] suffer from acoustic loss [43]. In [43] the deposition of parylene C on the upper surface before etching the back cavity allows to obtain a rigid-flexible coupling mechanism, which is able to maintain large vibration displacement of the unsealed membrane while avoiding acoustic loss.

As for microphones and speakers, it is possible to form arrays to improve the acoustic response and enlarge the bandwidth [89].

A summary of state-of-the-art MEMS speakers is reported in Table 2.

Typically, these devices cover the entire audio band; however, as their performance decreases for low frequencies, a few solutions focus more on enlarging the band at high frequencies [63]. Devices with a membrane area smaller than 2 mm^2^ are achieved for both electrostatic [47,83,86] and piezoelectric [40,59] actuation, while electromagnetic speakers, as expected, are bulkier. Driving voltages as low as 2 V peak-to-peak are achieved for piezoelectric speakers [40,46,63]. Acoustic responses larger than 90 dB SPL are measured at 1 kHz for all considered actuation principles. Piezoelectric solutions, in particular, may also achieve very high acoustic response (>80 dB SPL) over the entire audio band [35,46,62,89], while featuring low harmonic distortion [40,62] for-high fidelity sound reproduction.

With respect to MEMS microphones, which are already well spread commercially, MEMS speakers have not reached maturity yet and feature a significant room for improvement: MEMS piezoelectric devices, however, appear to be well suited for monopolizing the miniaturized speaker world in the future.

## 4. Interface Circuits

One of the main advantages of MEMS structures is the possibility to directly integrate the sensing or actuating device together with the required readout or driving circuitry. Interface circuit solutions for both MEMS microphones and speakers are discussed in this Section.

### 4.1. MEMS Microphones Interface Circuits

MEMS microphones are used in various applications, from personal electronics to computers, passing through automotive, peripherals, and high-fidelity (Hi-Fi) audio recording. This wide spectrum of implementations leads to a significant differentiation in the performance of microphone modules; indeed, distinct operating modes are necessary when the same device is utilized in systems with different specifications or when the specifications within the same system change according to the performed function.

**Figure 12:**
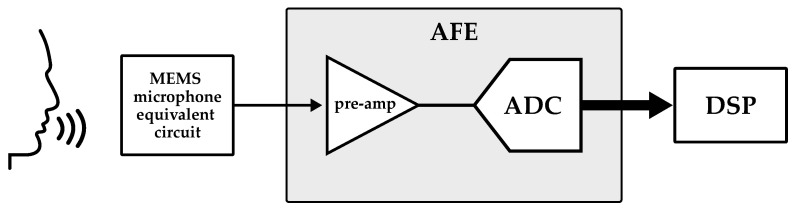
Block diagram of the readout chain of a MEMS microphone.

Although purely analog signal readout implementations are still used, most audio applications are digital. Accordingly, interface devices changed over the years from simple signal amplification circuits to complex mixed signal circuits: nowadays, in a typical audio chain, the electrical signal provided by the microphone is processed by an analog front-end (AFE) before being elaborated by a digital signal processing (DSP) block. The AFE consists of a pre-amplifier (pre-amp) and an analog-to-digital converter (ADC) circuit, as illustrated in Figure 12.

#### 4.1.1. Pre-Amplifiers

The typical range of environmental sound intensity is between 0 dB SPL (auditory threshold) and 140 dB SPL (threshold of pain). Since microphones’ sensitivity is usually around −45÷−35 dBV/Pa, this results into an electrical signal amplitude of only few mV (or tens of mV in the best cases), which is not strong enough for most applications. Audio pre-amps are therefore required to amplify this signal before feeding it forward; furthermore, they provide decoupling between the microphone and the rest of the circuit and allow a proper biasing of the microphone itself.

As previously discussed, the current state-of-the-art of MEMS microphones is strongly oriented towards capacitive and piezoelectric solutions, with a few instances of ElGoFET [22,75] and piezoresistive devices, while electromagnetic microphones are very rarely used. Consequently, pre-amp solutions that are well suited for capacitive, piezoelectric, piezoresistive, and ElGoFET microphones are discussed.

Independently from the microphone type, pre-amps can be divided into two main groups: variable gain amplifiers (VGA) or fixed gain amplifiers (FGA). VGAs, as the name suggests, can modify their gain in order to maintain the same output amplitude regardless of the input signal. This is especially useful when the ADC needs to work in a certain subset of its input range while minimizing distortion and noise [90]; in addition, it can also be used to reduce power consumption in specific time frames or even to enable different operating modes [91,92]. On top of that, other noise-cancelling techniques can be exploited, as shown in [93]. Gain variations can be implemented digitally (these amplifiers are also referred to as programmable gain amplifiers or PGAs) or exploiting analog control signals. FGAs, on the other hand, provide a constant amplification of the signal, thus resulting in less complex systems featuring lower power consumption and reduced silicon area.

Capacitive and piezoelectric microphones typically use the same pre-amp structures, either employing a constant-charge (CC) or a constant-voltage (CV) approach [94]; piezoresistive devices usually employ a resistive-bridge structure, while ElGoFET microphones adopt a specific readout architecture in order to sense the FET current. In addition, capacitive microphones require a charge pump circuit for providing the bias voltage to the sensor.

The CC approach is employed for capacitive and piezoelectric microphones when the latter feature a relatively high piezoelectric voltage constant with respect to the charge constant. Piezoelectric materials, indeed, are characterized both by a voltage constant and by a charge constant; the piezoelectric voltage constant is defined as the electric field produced in a material per applied unit of mechanical stress, while the charge constant is determined as the electrical polarization generated in a material per unit of applied mechanical stress [95]. A piezoelectric microphone where the voltage constant is dominant with respect to the charge constant substantially behaves as a capacitor, where the electrodes (top and bottom or planar and interdigitated) correspond to the capacitor plates. According to the CC approach, a constant charge is imposed on the capacitor plates; this can be achieved by charging the device to a fixed voltage during its fabrication and then ensuring good insulation. As the charge is fixed, when a sound pressure variation occurs on the MEMS device, a voltage fluctuation results from the changes in the capacitance.

**Figure 13:**
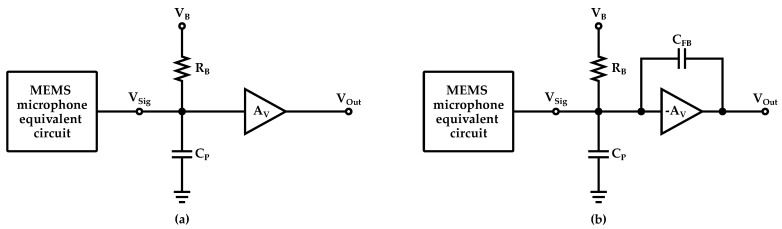
Schematic representation of a (**a**) CC approach and (**b**) CV approach based pre-amp circuit for MEMS microphones.

Voltage-to-voltage pre-amplifiers, illustrated schematically in Figure 13a, are used in this configuration. Particular attention should be paid to the biasing network at the $V_{sig}$ node since, to maintain the charge on the microphone, the pre-amp must feature high input impedance (tens of GΩ or more). The most utilized solution involves a relatively high resistance ($R_{B}$) which implements a high-pass filter with a cut-off frequency below the audio band frequencies (<20 Hz). Lastly, these amplifiers also need to ensure low output impedance to drive the following stages [93,96,97,98,99].

CV readout architectures are employed both for capacitive and piezoelectric microphones where the charge constant is dominant with respect to the voltage constant. A constant voltage is applied across the electrode plates, hence a sound pressure variation creates a charge signal proportional to the charge sensitivity of the microphone, which is the result of the product between the voltage sensitivity and the capacitance value in steady state. A charge amplifier scheme, such as the one illustrated in Figure 13b, is generally used to perform the charge-to-voltage conversion, thus ensuring low output impedance.

The advantage of this implementation is that the amplifier input is a low-impedance node, and therefore the signal voltage swing is quite small; this makes the parasitic effects of the capacitances that insist on this node negligible and relaxes the constraints on the biasing resistance, reducing the required value by at least two orders of magnitude. Even if this represents a significant advantage, the CV approach is not very popular for capacitive microphones, for which the CC approach is preferred. This is mainly for three reasons: in the first place, the output voltage with the CC solution only depends on the voltage sensitivity of the microphone, while in the CV scheme it also depends on the capacitance value in steady state, whose control during fabrication is poor; secondly, in the CV readout, the charge pump needed to bias the microphone has to provide current in order to generate the charge signal, whereas in the CC approach the charge pump only delivers current during system startup; and last, but not least, the CC approach is more versatile since it is adopted for the readout of a wide variety of other sensors (including electret microphones).

The CV approach is more common for piezoelectric microphones, as they typically employ materials, such as PZT, with dominant piezoelectric charge constants and can operate without a specific bias voltage, and thus without requiring a charge pump circuit, as they directly rely on mechanical pressure or vibration for the generation of an electrical signal.

**Figure 14:**
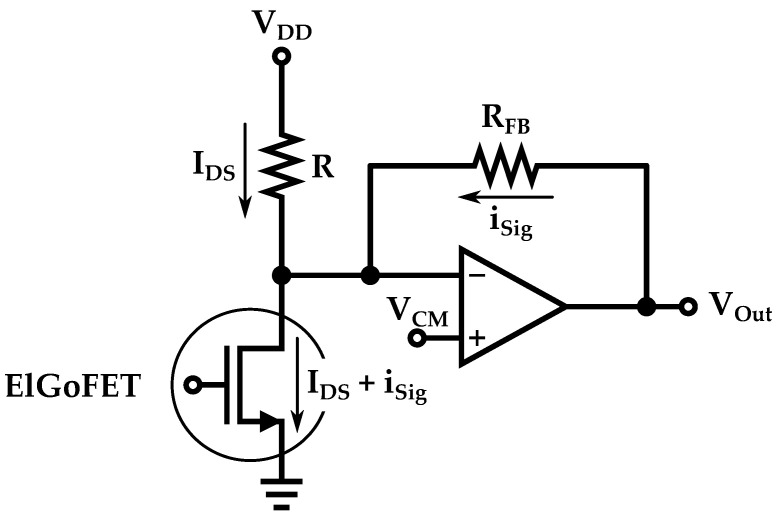
Schematic representation of the pre-amp circuit used as readout for an ElGoFET MEMS microphone. $V_{DD}$ is the supply voltage, $I_{DS}$ is the bias drain-to-source current of the FET device while $i_{sig}$ is the signal current.

ElGoFET microphones differ from traditional capacitive microphones as they employ a FET and sense sound by measuring the FET current variations determined by the diaphragm’s displacement. In order to measure the FET current a current-to-voltage converter architecture, as shown in Figure 14, is employed as a readout. As the FET drain terminal is connected to the inverting input of the operational amplifier, the FET operates under a fixed drain voltage condition for a given $V_{CM}$. The diaphragm displacement produces variations in the electric field of the gate oxide, which gives rise to a signal current flowing through the feedback resistor ($R_{FB}$) that, consequently, results in a pressure-dependent voltage signal at the output of the operational amplifier [75].

**Figure 15:**
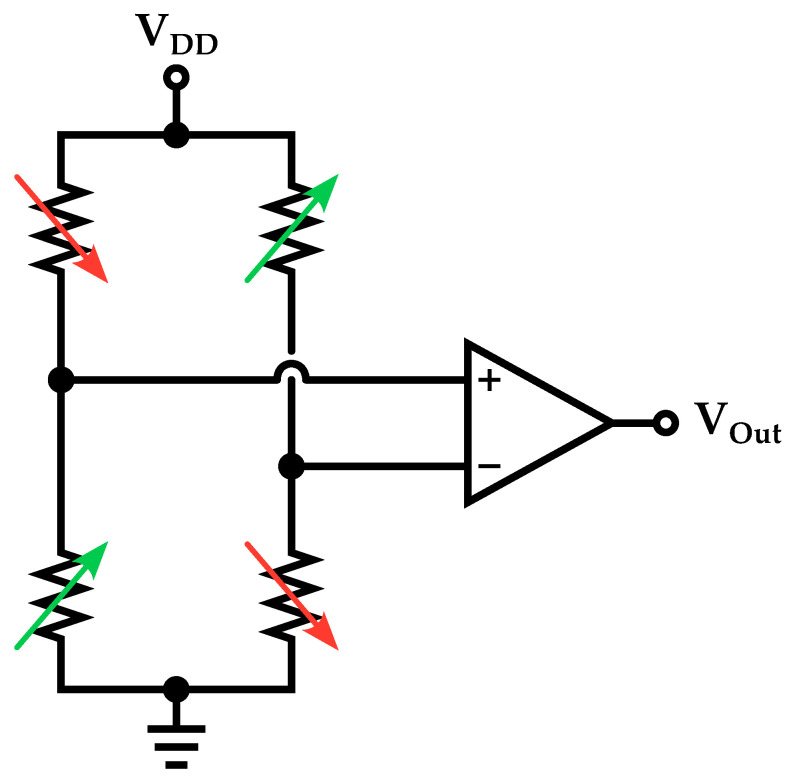
Schematic representation of the pre-amplifier circuit for a piezoresistive MEMS microphone.

In piezoresistive microphones, sound pressure determines resistance variations; hence, the microphone interface circuit substantially consists of a resistive sensor readout. Typically, this kind of devices consists of four piezoresistors, which are arranged in a Wheatstone bridge configuration [20,100], and exhibit a variation based on the applied stress determined by the sound waves. The piezoresistors are typically designed so that, upon the occurrence of sound pressure, two will be compressed while the other two will be stretched, thus producing a differential signal: this technique ensures self-cancellation of random accelerations [100] improving the performance of the readout. Typically, the differential voltage signal is then processed employing an instrumentation amplifier as pre-amp. With respect to piezoelectric microphones, piezoresitive ones require a direct and stable biasing of the device. A schematic representation of the Wheatstone bridge and pre-amp structure is reported in Figure 15.

#### 4.1.2. Analog-to-Digital Converters

After being processed by the pre-amp, the analog audio signal needs to be converted into the digital domain; it is undoubtedly easier to work with bits when it comes to manipulating and extracting information, as digital signals have the capability to transmit information with reduced noise, distortion, and interference. Furthermore, they can also be stored for later utilization.

Resolution is a key aspect in the analog-to-digital (A/D) conversion framework: the higher it is, the more information can be conveyed within the digital signal. Reducing the quantization error allows the performance high-level bit manipulation. Considering, as an example, an audio signal that contains specific sounds and environmental noise, with a 5 bits resolution it is possible to detect the presence or the absence of audio power above the noise floor while with 8–10 bits, not only the power can be discerned, but it is also possible to recognize the type of sound (e.g., words or melodies). Moreover, with 12 or more bits, more complex applications can be implemented since the digital signal can be fed to neural networks or artificial intelligence (AI) systems.

Among ADC types, the ones that can reach relatively high resolution are oversampling converters. This type of ADCs is an excellent option for audio applications because the limited bandwidth ($BW_{audio}=$ 20 ÷ 20,000 Hz) enables the use of high oversampling ratios (OSR) without the risk of encountering excessive clock frequencies. Sigma-Delta (ΣΔ) converters are widely used thanks to their low power consumption and inherent linearity; especially suited for low-frequency applications, they can reach very high SNR values with simple hardware at the expense of speed. In general, for a *L*th order ΣΔ modulator based on an N-bit quantizer and having OSR equal to *M*, according to [101],

(1) $$SNR=\frac{2^{2N}3\left(2L+1\right)M^{2L+1}}{2\pi^{2L}}.$$

ΣΔ modulators can be classified into two main categories: discrete-time ΣΔ modulators and continuous-time ΣΔ modulators. Discrete-time ΣΔ modulators operate on signals that are sampled at discrete time intervals and are processed digitally; these modulators typically include an ADC that samples the input signal at a high frequency, followed by a digital filter and a digital-to-analog converter (DAC) [102,103]. In continuous-time ΣΔ modulators, the input signal is sampled continuously and processed in the analog domain; it is constantly compared to a reference voltage, determining a sequence of decisions corresponding to a bitstream. This data is subsequently low-pass filtered and decimated to obtain the final digital output [104,105,106,107,108,109,110]. Ideally, continuous-time ΣΔ modulators are more power efficient than discrete-time ones, but, on the other hand, they are more sensitive to process variations and clock jitter.

A topology that has gained popularity in recent years is the Noise-Shaping Successive Approximation Register (NS-SAR) ADC. This converter architecture offers the advantages of a SAR ADC, such as low power consumption, high conversion efficiency, and small area occupation, while also providing the benefits of noise shaping, which greatly enhance the overall resolution; moreover, unlike oversampled ΣΔ converters, this hybrid topology is well-suited to scale with technology. These converters modify the spectral shape of the quantization error, causing its contribution to be pushed to a higher frequency outside the audio band, rather than being uniformly distributed. By combining this effect with oversampling techniques and filtering the out-of-band spectral components, the effective number of bits (ENOB) can be considerably increased. Examples can be found in [111,112,113,114].

### 4.2. MEMS Speakers Interface Circuits

MEMS Speakers are driven by means of Power Amplifier (PA) circuits. The audio PA receives an electrical signal as input and delivers it, amplified, to the MEMS speaker, which converts it to an audible signal. The input signal can be either analog or digital, while the output signal has to be analog in order to be audible to human ears.

In order to evaluate its performance and, therefore, specify the circuit requirements, an audio amplifier can be characterized by looking at a few significant parameters: Total Harmonic Distortion (THD), SNR, efficiency and Power Supply Rejection Ratio (PSRR). THD measures the amount of distortion introduced into the audio signal by the amplifier: lower THD values are better, as they indicate that the amplifier is generating a more faithful reproduction of the original audio signal; typical THD values are around 0.01–0.1% [115,116,117,118,119,120,121]. SNR measures the ratio of the desired audio signal with respect to unwanted noise introduced by the amplifier; higher SNR values are desired, as they indicate that the amplifier is producing a cleaner and more accurate audio signal; usually SNR exceeding 100 dB are pursued [119,120,121]. The efficiency of an audio amplifier is an important parameter since it determines how much power is wasted as heat and how much is delivered to the load; typically, efficiency values larger than 90% are desired. Last but not least, PSRR measures how well an amplifier can reject noise and other unwanted signals present in its power supply; indeed any noise or fluctuations in the power supply can introduce unwanted artifacts into the audio signal, leading to distortion at the output; for this reason, PSRR values larger than 50 dB are required [115,119,122].

**Figure 16:**
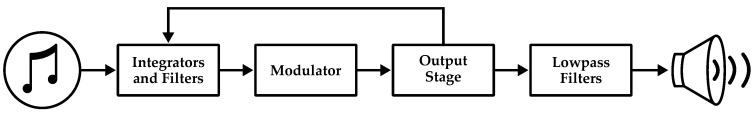
Block diagram of the driving chain for a MEMS speaker.

One of the most common amplifier architectures for MEMS audio applications is the class D amplifier. Unlike traditional analog amplifiers, which employ linear amplification to increase the voltage or current of the audio signal, class D amplifiers use pulse-width modulation (PWM) to represent the audio signal. This allows to achieve much higher efficiency, typically exceeding 90%: in this way, they generate less heat and can be made smaller and lighter. Despite these advantages, they can be complex to design and manufacture and may also require sophisticated components. The class D amplifier scheme, indeed, consists of several blocks: integrators and filters, modulator, output stage, and lowpass filters, as illustrated in Figure 16.

**Figure 17:**
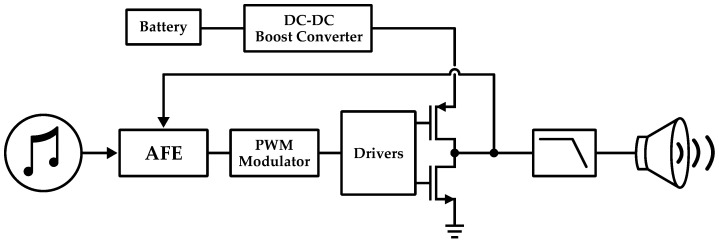
Schematic representation of a standard class D amplifier for audio applications.

The integrator and filters provide high gain and stability to the loop while the modulator generates a signal to be amplified by the output stage. The most common modulation technique is the previously mentioned PWM but other methods are also possible: bang-bang control [115], delta-sigma [116] and self-oscillating [123]. The output stage is responsible for taking the modulated signal and amplifying it to a level suitable for driving the MEMS speaker. It is typically realized using a pair of transistors arranged in a half-bridge configuration: in this way the voltage applied to the load, i.e., the MEMS speaker, switches between two levels, usually ground and the supply voltage, at a high frequency. The output filter of a class D amplifier is typically a low-pass filter that removes the high-frequency components of the PWM delivering a high-fidelity audio signal to the speaker. A feedback is also present for improving the overall performance of the amplifier. Some examples of class D amplifiers can be found in [117,118,119,124] while a schematic representation of it is illustrated in Figure 17.

Alongside class D, class AB amplifiers are also commonly used for audio applications. They lie in between class A and class B, offering an efficiency of about 60%, which is significantly worse than class D but, generally, feature lower distortion, resulting in a higher fidelity sound, as presented in [122].

With the evolution of MEMS speakers, a larger output power is required; it can be achieved by increasing the voltage swing at the amplifier output, e.g., by boosting the supply voltage. Accordingly, DC-DC converters can be employed for driving the amplifier; one of the most common ones is the boost converter. Boost converters are used when the input voltage is lower than the required output voltage of the amplifier; they are a popular choice for driving audio amplifiers due to their high efficiency and ability to provide a regulated output voltage that is suitable for driving a wide range of audio devices. Some examples of boost converters for driving a PA are shown in [120,121]. The boost converter is not the only DC-DC converter that can be employed for this purpose; overall, the choice and features of the DC-DC converter to be selected depend on the specific requirements of the application and the desired performance of the PA and of the MEMS speaker.

## 5. Conclusions

This review provided a complete analysis of the world of MEMS audio devices, discussing their field of application, the transduction principles they rely on, the state-of-the-art scenario and the architectures employed for implementing their interface circuits. Microphones have been recognized as the driving force of MEMS audio devices, however speakers are expected to experience a boom in the coming years; indeed, while MEMS microphones have been the object of intensive research since the early 2000s, research has focused on speakers only more recently. Although electrostatic transduction has been the method chosen for the majority of commercially MEMS microphones, and hence MEMS audio applications, the trend for the future of both sensing and actuating devices appears to be more oriented towards piezoelectric solutions. Indeed, piezoelectric devices feature the advantage of very low power consumption, are well suited to miniaturization and may exploit the interface circuits already developed for electrostatic microphones and electromagnetic speakers. Furthermore, their drawback, i.e., featuring decreased performance, is being addressed and possibly solved by the current advancements in process technologies and material properties. 

## Figures and Tables

**Table 1:** Summary of state-of-the-art MEMS microphones.

	Type	Membrane Size [mm^2^]	Sensitivity * [dbV/Pa]	Bandwidth [Hz]	SNR [dB]
[70] (2007)	Capacitive	4	−43.4	30–20,000	/
[32] (2009)	Capacitive	0.25	−74	1–20,000	/
[33] (2010)	EM	201	−54.8	50–20,000	/
[4] (2011)	Capacitive	0.785	−38	60–20,000	32.03
[75] (2015)	Capacitive ElGoFET	1.131	/	50–20,000	/
[11] (2017)	Capacitive	0.196	−37.1	20–20,000	/
[5] (2017)	Piezoelectric AlN	2.01	−63.4	20–10,000	/
[6] (2017)	Capacitive	0.071	−64	200–10,000	/
[57] (2017)	Capacitive	0.454	−45.5	562–2200	45
[80] (2017)	Capacitive	3.64	−38.7 ^†^	100–10,000	/
[71] (2018)	Capacitive	0.09	−52.1	1–20,000	/
[7] (2018)	Capacitive	0.503	−39.8	50–22,000	54
[81] (2019)	Piezoelectric AlN	1.947	−45.3	20–20,000	62.65
[18] (2019)	Piezoelectric ZnO	/	−17.2	50–1200	85.8
[79] (2019)	Piezoelectric AlN	0.88	−49.3	1000–20,000	64.65
[72] (2019)	Capacitive Graphene	9.621	−74	100–20,000	/
[23] (2019)	Capacitive	0.503	−40.5	50–20,000	57.8
[13] (2020)	Piezoelectric AlN	4.909	−47	2000–10,000	67.03
[22] (2020)	Capacitive ElGoFET	/	−52.4 ^‡^	5–500	/
[76] (2020)	Capacitive	1	−35.9	100–4900	/
[77] (2020)	Piezoelectric Custom PZT	/	−37.5	20–20,000	48.9
[19] (2020)	Piezoelectric ZnO	6.25	−17.6 ^†^	100–1000	/
[31] (2020)	Piezoelectric ZnO	6.25	−13.9 ^†^	856–8820	/
[24] (2021)	Piezoelectric Mo/AlN	4.99	−23	300–8000	51
[82] (2021)	Capacitive	/	−34	100–20,000	73
[78] (2021)	Piezoelectric PZT	0.64	−33.2	20–20,000	82.4
[20] (2022)	Piezoresistive	/	−43.3	20–2000	38.6

* At 1 kHz unless otherwise specified. ^†^ At resonance. ^‡^ Minimum value across the whole bandwidth.

**Table 2:** Summary of state-of-the-art MEMS speakers.

	Type	Membrane Size [mm^2^]	Driving Voltage	Acoustic Response [dB SPL]	Acoustic Response across Bandwidth [dB SPL]	Bandwidth [Hz]	THD
[49] (2008)	EM	4.909	/	106	/	/	/
[48] (2011)	EM	9.62	/	106	>70	125–8000	/
[42] (2013)	EM	176.71	/	80	>80	330–70,000	/
[61] (2015)	Piezoelectric PMN–PT	50.27	5 V_RMS_	66.2	>47	100–10,000	/
[83] (2015)	Electrostatic	0.25	8 V_DC_ + 6 V_AC_	55.56 *	/	/	/
[84] (2016)	Electrostatic	12.5	10 V_DC_ + 1.9 V_RMS_	35 ^†^	>5	3000–20,000	/
[34] (2018)	EM	9.62	/	90	>40	10–10,000	/
[46] (2018)	Piezoelectric PZT	16	2 V_PP_	90	>81	20–20,000	<7%
[40] (2020)	Piezoelectric Thin Film PZT	1	2 V_PP_	79.5	>50	20–20,000	<2%
[39] (2020)	Piezoelectric Ceramic PZT	28.27	10 V_PP_	80	>60	100–10,000	/
[51] (2020)	Piezoelectric Sc-doped AlN	9	20 V_DC_	/	>50	20–20,000	/
[86] (2020)	Electrostatic	1.887	30 V_DC_ + 30 V_AC_	60	>55	200–20,000	/
[63] (2021)	Piezoelectric Thin Film PZT	2.54	2 V_PP_	92 *	>45	14,000–30,000	/
[43] (2021)	Piezoelectric PZT + Parylene	/	2 V_DC_	101.2 *	>53	20–20,000	/
[59] (2022)	Piezoelectric AlN	1.96	10 V_RMS_	73	>73	100–10,000	<4%
[35] (2022)	Piezoelectric AlN	16	8 V_RMS_	104	>80	20–20,000	<5%
[88] (2022)	Piezoelectric PZT	16	5 V_PP_	85	>60	20–20,000	<5%
[62] (2023)	Piezoelectric sol-gel PZT	4	10 V_DC_ + 0.7 V_RMS_	85	>80	20–20,000	<7%
[89] (2023)	Piezoelectric Film PZT	7.5	3.5 V_RMS_	99	>81	60–15,000	/
[36] (2023)	Piezoelectric AlN	/	10 V_RMS_	95	>75	20–20,000	<2.5%

* At resonance. ^†^ At 20 kHz.

## Data Availability

No new data were created or analyzed in this study. Data sharing is not applicable to this review.

## Abbreviations

The following abbreviations are used in this manuscript:

ADC	Analog-to-Digital Converter
AFE	Analog Front-End
AI	Artificial Intelligence
AR	Augmented Reality
CAGR	Compound Annual Grow Rate
CC	Constant-Charge
CMOS	Complementary Metal Oxide Semiconductor
CV	Constant-Voltage
DAC	Digital-to-Analog Converter
DSP	Digital Signal Processing
ElGoFET	Electret Gate of Field-Effect Transistor
EM	ElectroMagnetic
ENOB	Effective Number Of Bits
FET	Field-Effect Transistor
FGA	Fixed Gain Amplifier
HMD	Head Mounted Display
IC	Integrated Circuit
IoT	Internet of Things
MEMS	Micro-Electro-Mechanical System
NS-SAR	Noise Shaping Successive Approximation Register
OSR	OverSampling Ratio
PA	Power Amplifier
PDMS	PolyDiMethylSiloxane
PGA	Programmable Gain Amplifier
PSRR	Power Supply Rejection Ratio
PWM	Pulse-Width Modulation
SEM	Scanning Electron Microscope
SiNW	Silicon NanoWires
SNR	Signal-to-Noise Ratio
SPL	Sound Pressure Level
THD	Total Harmonic Distortion
TWS	True Wireless Stereo
VAD	Voice Activity Detection
VGA	Variable Gain Amplifier
VR	Virtual Reality
